# Perceived implications of COVID-19 policy measures on food insecurity among urban residents in Blantyre Malawi

**DOI:** 10.1186/s12889-022-12922-6

**Published:** 2022-03-17

**Authors:** Mastano N. Dzimbiri, Patrick Mwanjawala, Emmanuel Chilanga, George N. Chidimbah Munthali

**Affiliations:** 1 College of Education, Health and Society, Miami University, 2007McGuffy Hall, Oxford, OH USA; 2 Department of History, Miami University, Oxford, USA; 3 School of Social Work, McGill University, Montreal, Canada; 4 School of Economics and Management, Yangtze University, Jingzhou, China; 5 Mzuzu University, Mzuzu, Malawi; 6 Chidimbah Research Centre, Luwinga, Malawi

**Keywords:** Food insecurity, Urban workers, COVID-19 policy, Vulnerability, Urban residents

## Abstract

**Background:**

Malawi is at the brink of experiencing food insecurity amidst the COVID-19 pandemic as the vast majority of its population lives in extreme poverty. While measures are being implemented to avert the spread of COVID-19, little is known about how COVID-19 policy measures have impacted food insecurity in urban Malawi. This study addresses this gap by exploring the implications of COVID-19 policy measures on food insecurity in low-income areas of Blantyre in Malawi.

**Methods:**

We used Bronfenbrenner's ecological theory to explore the implications of COVID-19 policy measures on peoples’ access to food. In-depth interviews were conducted with fifteen participants comprising of private school teachers, street vendors, sex workers, and minibus drivers. Data were analyzed using thematic analysis in which emerging patterns and themes from the transcripts were identified.

**Results:**

The COVID-19 lockdown measures undermined participants’ ability to maintain livelihoods. These measures have increased the vulnerability of the residents to food insecurity, forcing them to face severe challenges to accessing adequate food to support their families as a result of low incomes, job loss, and business disruptions.

**Conclusion:**

Our study underscores the need for the Malawi government to seriously consider the provision of basic necessities such as food to the urban poor. We also suggest that the Malawi government should continue and expand the social cash transfer or relief funding packages by targeting the most vulnerable groups in the city. There is also a need for the government to engage all stakeholders and work collaboratively with people at local level in policymaking decisions in times of crisis.

## Introduction

As the world continues battling with COVID-19, which has seriously disrupted the global economy as well as the food supply chain, several countries are at risk of being food insecure [1]. Food insecurity is defined as the inability to access nutritious, safe food as well as feeling not being satisfied with the food consumed [2]. A recent report by the World Food Program shows that in the year 2020, about 137 million people worldwide faced acute food shortages, and this figure is significantly higher compared to the initial estimates before the inception of COVID-19 [3]. The United Nations Sustainable Development Goals set an ambitious goal–-to end hunger by 2030. Unfortunately, the pandemic has disrupted this goal, as over 150 million people globally today are stuck in extreme poverty [4]. A report released by the World Bank predicted that nearly 40 million people in Sub-Saharan Africa would be pushed into extreme poverty as a result of the impact of the COVID-19 by the end of 2020 [5]. Malawi remains one the poorest countries in the world with more than half of the country’s population living below the poverty line (less than $1.90 per day), and a quarter living in extreme poverty [6]. An increase in the levels of poverty has been historically driven by low agricultural productivity exacerbated by catastrophic weather events and the outbreak of COVID-19 has disproportionately resulted in widespread food insecurity [7]. Perceptions of food security and insecurity in Malawi center around maize production, distribution, and utilization. While Malawi produces a variety of food crops, maize accounts for 90% of the produce. It is the staple food in the country, consumed by the entire population. Other crops such as rice and potatoes only supplement maize consumption. Thus, in a Malawian context, the availability of and access to sufficient maize defines food security [8].

Given the already existing problems of extreme poverty, hunger, water shortages, and the burden of diseases affecting Sub-Saharan Africa (SSA), the COVID-19 pandemic has worsened the problems. There is inadequate knowledge regarding the questions of who and how COVID-19 policy measures threaten food security among the urban poor in SSA countries such as Malawi. In Malawi, the first case of COVID-19 was registered on April 2, 2020 [9]. As of January 2021, Malawi had about 14,851 COVID-19 cases and 353 deaths [10]. This included the demise of high profile and widely known politicians as well as Government officials such as Sidick Mia who was the Minister of Transport as well as Lingson Berekanyama who was the Minister of Local Government [7]. As COVID-19 cases and related deaths surged tremendously, the Malawi Government, under the leadership of Lazarus Chakwera, desperately enacted strong restrictive policy measures as a way of containing the spread of the virus. Some of the measures implemented included: (i) reducing the capacity or number of passengers boarding minibuses and taxis (ii) closing bars and marketplaces at eight o’clock in the evening time, and (iii) closure of schools, and social distancing [7]. These measures were deemed to be extremely harsh by the urban dwellers especially those in the informal work force such as street vendors, sex workers, and minibus/tax drivers, as they posed a threat to their livelihoods [10]. Thus, sex workers and minibus drivers across the country took to the street demonstrating against the COVID-19 policy measures. Studies have documented the impact of COVID-19 on health, agriculture, and the economy in general, but little has been researched to explore how COVID-19 policy measures have affected informal and private school teachers in urban Malawi.

Therefore, our study addresses this dearth in knowledge by exploring the perceived implications of the COVID-19 policy measures on food insecurity among the low-and unstable income residents in the urban areas of Blantyre in Malawi. Understanding the nexus between COVID-19 measures and food security in the Malawian context is crucial for effective implementation of interventions that can help reduce the risk of being food insecure while preventing further spread of the virus. Food insecurity is linked to child stunting, and this remains a problem in Malawi as evidenced in the central region [11]. Our study contributes to the existing literature by exploring the emerging geographies of food insecurity linked to COVID-19 by answering the following questions: i) What are the perceived experiences of food security amidst COVID-19 policy measures? and ii) How have COVID-19 policy measures affected access to food and food consumption patterns? These questions helped us to address our goal by documenting who and how the COVID-19 policy measures have impacted on food security among the urban residents in Malawi’s secondary city of Blantyre.

### Conceptual framework

We used Urie Bronfenbrenner’s ecological theory to better understand the implications of COVID-19 policy measures on food security among low- or unstable-income residents in Malawi’s secondary city of Blantyre. Bronfenbrenner’s ecological theory unfolds the interconnectedness between the environment and the people it nurtures in the context of accessing basic resources. Thus, the theory stresses that external factors can influence an individual’s capacity to do certain actions, and at the same time, the environment can be altered by the individual’s activities. External factors can include but are not limited to interpersonal, societal, community, organizational, state, and/or national levels. These are nested within the broader context of culture and policies that reinforce specific values and individual behaviors. Several studies have been conducted globally to examine issues of food security as well as disease burdens using a socio-ecological framework.

For instance, a study that assessed factors influencing the consumption of fruits and vegetable using a socio-ecological framework among adolescents in Uganda found that background factors, individual factors, knowledge, attitudes, and beliefs were the key drivers of fruit and vegetable consumption among adolescents [12]. Likewise, a study that explored the barriers that influence the adoption, sustainability, and consistent use of sanitation facilities using a socio-ecological framework revealed that a complexity of multiple factors ranging from individual, household, community to national levels had implications on the adoption and use of sanitation facilities in rural areas of Ethiopia [13]. In this instance, our application of ecological theory helped us to understand how the societal systems and structures such as government policies on COVID-19 have exacerbated household food insecurity by looking at changes in daily meal consumption as well as people’s capacity or ability to access adequate foods.

## Methods

### Study locality

**Fig. 1:**
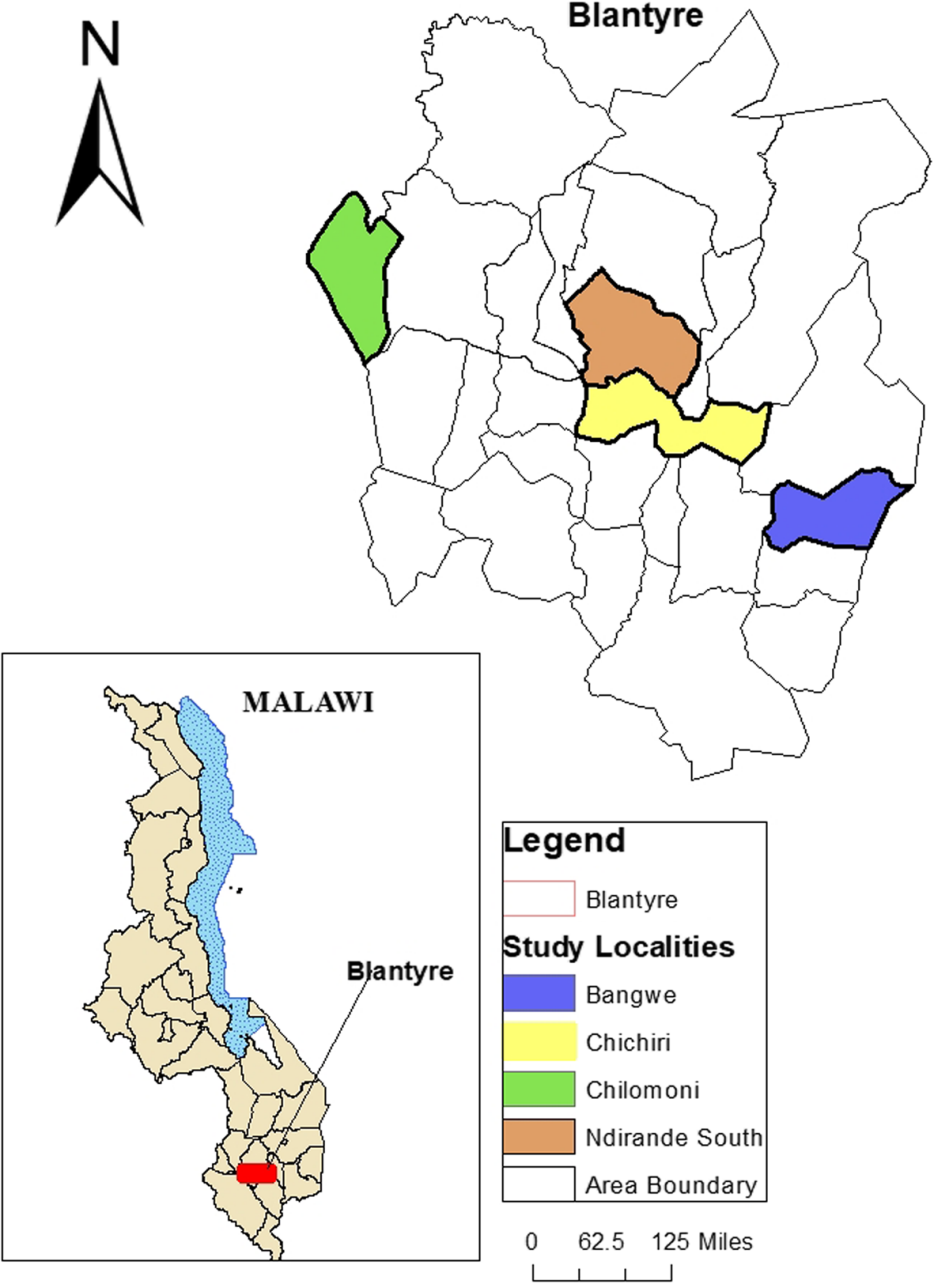
Study localities within Blantyre city Malawi (created by authors in ArcMap version 10.16)

Malawi is located in Southern Africa, bordered by Tanzania to the north, Zambia to the west, and Mozambique to the south (Fig. 1). The National Statistical Survey Report of 2017 shows that there were about 17.6 million people in Malawi. More than half of the country’s population is living below the poverty line and a quarter is trapped into extreme poverty. Malawi has three main cities: Mzuzu, Lilongwe, and Blantyre, situated in the North, Central, and Southern regions, respectively. This study was conducted in Blantyre city, one of the oldest cities in Malawi [8]. Our study focuses on an urban area for the following reasons. First, there has been an increase in the number of COVID-19 cases, which have disproportionately increased urban poverty as many people have lost their jobs due to closure of some industries as well as disruption of business activities [6]. Second, based on our knowledge, the lockdown and many other restrictive measures were mainly applied in urban areas where people survive through working, unlike in rural areas. Given that Blantyre city serves as the second largest commercial city in Malawi, informal employment is the main source of income for the vast majority [8]. Most people are engaged in agricultural activities, and unregistered work such vendors, sex workers, domestic workers, and hawkers [14].

### Data collection and analysis

We recruited a research assistant who was fluent in both English and Malawi’s local language, Chichewa, to collect data between December 2020 to April 2021. The research assistant was trained in qualitative and survey studies, research ethics, and all research protocols regarding the sensitivity of the topic under study in relation to the nature of the participants. Besides, the research assistant is a university graduate and has over 5 years of experience working in qualitative research with local organizations in Malawi. We interviewed fifteen participants (*n* = 15) comprising of minibus drivers, street vendors, private secondary school teachers, and sex workers. We targeted these participants based on their low socio-economic class status, as most of them have unstable or unreliable sources of income. Participants were drawn from different localities within the city region of Blantyre—namely Bangwe, Chichiri, Chilomoni, and Ndirande south (Fig. 1). For ethical reasons, all the names assigned to participants in this manuscript are pseudonyms. Prior to the actual interviews, participants were asked if they reside in Blantyre city, which served as an inclusion/exclusion criterion for recruiting the participants. Participants’ age ranged from 18 to 31 years old, and they had a family size of five members on average. We determined the sample size and the participants based on the principle of maximum variation to collect diverse data [15]. A snowball sampling strategy was used to identify the participants in which a referral was obtained from the first participants to get the next one who shared similar socio-economic characteristics or social network [16]. Interviews were conducted to capture in-depth information regarding their experiences about feeling food insecure as a result of COVID-19 preventative measures.

The interviews with some participants were conducted within the heart of the city of Blantyre, while other respondents were interviewed in their residential areas (Fig. 1). We observed social distancing and face mask covering measures during the interviews. The interviews lasted between 40–90 min. In qualitative studies, interviews are usually conducted to a small number of people with an aim of capturing individuals’ views and understanding about a certain phenomenon [16]. Interview guides containing modified questions adopted from a Household Food Insecurity Access Scale (HFIAS) were sent to our research assistant in Malawi. The questions adopted and modified revolve around the following key constructs of HFIAS: (i) participants’ feelings of uncertainty over food access, ii) perception of having inadequate food, iii) reduction in food intake/consumption, and iv) consequences of the reduction in the intake of food [17]. These constructs were used as a proxy indicator to assess food security. A consent to participate in the study was obtained from each participant verbally in either English or Chichewa languages.

Data were transcribed verbatim from Chichewa, the local language, to English. We used the five steps of qualitative data analysis [16]. First was data immersion, whereby all study authors [4] independently read the transcripts more than five times. The field notes (memos, observation notes and photos) were also independently read and viewed. We discussed our understanding of the content of the transcripts and agreed to continue with the analysis independently. The second step involved coding of the transcripts. We had the first circle of coding whereby each one of the authors identified between 16 to 19 codes. The third step involved generation of common categories. In this process, authors discussed the common codes and came up with 7 categories. The last stage involved data reduction where three themes were mutually generated. These are people’s access to income amidst COVID-19, access to food, and changes in eating habits.

These themes have been presented and elaborated in the results section. Direct quotations from the respondents were presented narratively, in the form of stories. All the methods presented in this study were done following the institutional guidelines and regulations as detailed in the ethics approval letter.

### Research ethics review

Ethics approval to conduct this study was obtained from the University of Livingstonia research ethics committee in Malawi by the third author (protocol number: UNILIA-REC/1/CUP 2/01). We also sought written permission from the Blantyre City Assembly. We also obtained written consent from the participants prior to the interviews.

## Results

This section presents the results, which show how COVID-19 policies have aggravated food insecurity in Malawi’s Blantyre city. We interviewed fifteen participants, reviewed the findings, and crafted our work under the framework of ecological theory, reflecting on how the changes in the government’s policies have concurrently altered food consumption and access. Our results revealed that residents in Blantyre city are facing significant challenges to accessing adequate nutritious food due to the policy measures against the spread of the virus. Our findings are categorized into the following themes: people’s access to income amidst COVID-19, access to food, and changes in eating habits.

### Access to income amidst COVID-19

The awareness of the COVID infection has directly lowered people’s accessibility to financial resources [18]. In regard to our study, various people interviewed complained about poor economic performance as a product of the fear of COVID infection. As generally articulated, social gatherings and personal contacts can spread the coronavirus within a split second. This knowledge and fear discouraged people from accessing the sex workers’ services. A thirty-one-year-old sex worker confirmed the detachment of existing clients by articulating that:

“The nature of our job puts us and our customers at risk of contracting the virus, and due to this, we have lost several valued and potential customers”*.* (Goweko, Sex worker)

Losing customers reduced the financial or income status of the sex workers who depend on the provision of services to feed their families. Stories of sex workers resonate with many citizens who depend on informal work in the city. Street vendors shared similar stories as buyers of their items were unlikely to present themselves in crowded towns, fearing COVID-19 contraction. This bears profound consequences on the outcome of business in town. The vendors profit less than usual from the sales, hence affecting their access to financial wealth and resources. One of the vendors articulated that:

“We used to make a lot of money before the implementation of the Covid-19 restrictive measures such as the curfew and closing the marketplaces exceedingly early. You know here in Malawi, business activities such as selling items like fish, vegetables, tomatoes, rice, etc. take at its peak from 7pm to late night hours. So, these restrictions have heavily disrupted our usual businesses activities and we are not making enough money at all” (Segula, street vendor).

Similar concerns were also raised by minibus/taxi drivers. Most of them complained heavily about the government decision in reducing the capacity of the passengers boarding either minibuses or taxis as one of the measures in place to reduce the spread of the coronavirus. According to the participants, reduced carrying capacity of the passengers means a cut of or reduction in income made per trip. Thus, drivers reported that they, due to reduction in the number of passengers, received less earnings per day as most of them could not make enough money. One of the drivers reported that:

“I am very much concerned and worried about myself and the future of my family. I am not making enough money nowadays as I used to in the past. I used to have a lot of customers per day and made more money by the end of the day, but now it is not happening anymore. I am now struggling to support my family and pay rentals bills because my income is not that good” (Thomas, driver).

### Food access during the COVID-19 pandemic

The measures against further spread of COVID-19 suppressed people’s accessibility to food. During interviews, participants reported that the government, in response to the COVID-19 outbreak, instituted a curfew, commencing at 8 pm of every single evening. This measure required the urban dwellers to close businesses and desert the town at the demanded time. However, informal businesses often receive more customers during the evening hours than during the daytime. Because of the curfew, informal businesses received fewer customers and lost income. In our interviews, sex workers reported to face income depreciation with the installation of the curfew. One of the sex workers interviewed said that:

We used to make a lot of money in night clubs and bars just by entertaining or dancing for the clients but now with the government's restrictions, all these entertainment centers are now running for a few hours and it is extremely hard to make a reasonable amount of money as we used to make before (Chamoka, Sex worker).

In accordance with the above views, another sex worker expressed her concern by overwhelmingly saying that:

“We can’t find money that could have been used to buy healthy food because our clients are scared to come out of their houses at night.” (Hawaya, Sex worker).

Feeling discontented with this development, sex workers marched in the streets, demanding the government to undo the curfew. Most vendors who depend on informal business also suffered the consequences of preventive measures, alongside sex workers. With the curfew in towns, business remained open only during unprofitable hours and shut down at the time people are available to purchase food items.

Additionally, the people’s purchasing power decreased, worsening profitability at the market. The reduction in purchasing power originated from the closure of schools, which downsized the income of the teachers by up to 100%.

Private schools entirely survive on tuition fees collected from students. When the government shut down schools to disperse the students away from each other, a collection of schooling fees to pay teachers also shut down. In the end, teachers only walk home with little or nothing, and this hugely impacts food access and consumption. One private school teacher complained:

“Some of us do not have enough money to buy proper food due to the closure of schools. The owners of the schools argue that they cannot pay us our usual salaries because we are not working as the schools are closed in compliance with the government policies” (Kapadala, Teacher).

Vendors face similar limitations to access food for their daily survival as stated:

“I am not making as much money as I used to make before, and this has resulted in me failing to afford buying enough relish or maize flour that could last me and my family for the whole month.” (Mnatharu, Street vendor).

### Changes in eating habits

Residents in Blantyre city have changed eating patterns just to prolong their survival and reduce sufferings resulting from limited access to food resources. During interviews, participants reported they invented ways of mitigating the effects of food inaccessibility as a result of the pandemic.

They opted to eat less than usual. When asked the meaning of this phrase, participants referred to skipping one meal as well as preparing only half of the normal size of the food consumed. In Malawi, people usually follow a meal plan of three meals that commences with breakfast, lunch and then dinner.

However, the outbreak of COVID-19 has crippled food accessing power, limiting the quantity and quality of the food available for consumption. In response to this challenge, citizens resigned to skipping meals as a way to extend consumption of the little food available at home.

Apart from adjusting to eating only twice a day, they also ate a smaller portioned meal. One of the private school teachers enriched our understanding by making these remarks:

“During this time while we are waiting to be called back at work, we have to eat less in order to have little money to buy food in the near future in case you are at home for a longer time without a job”. (Mr. Dokiso, teacher)

Eating less and skipping meals have become the possible strategies of combating the pressure of food inaccessibility and also the normal eating habits during the pandemic. We note that people also normalize eating less nutritious food as part of the food coping strategy during the reign of the pandemic. Foods rich in nutrients cost higher than less nutritious ones. In Malawi, meat, chicken, fish, and other proteins are more expensive than vegetables, sweet potatoes, cassava, and soya beans. With shortages in household incomes, diversifying food consumption to ensure maximum utilization of nutrients becomes an obstacle. A private secondary school teacher reported that:

“I am not earning my full salary and due to this I am not able to buy various kinds of foodstuffs because the money is not enough, and things are now expensive”*.* (Asamma, Teacher).

Therefore, people eat what they can afford, usually cheap and less nutritious food. In accordance with the perspectives of the above teacher, one of the sex workers articulated these words during the interviews:

“Due to little or no money, I have been finding problems buying and eating healthy food. I mainly resort to anything that is extremely cheap” (Chisomo, sex worker).

## Discussion

This study employed a purely qualitative inquiry approach by conducting in-depth interviews to explore and better understand how COVID-19 policy measures have impacted food insecurity among urban residents in Blantyre city, southern Malawi. We crafted our discussion of the findings within the lens of Urie Bronfenbrenner's ecological theory by demonstrating how macro-level policy decisions affect people at the household or community levels. Our study findings are consistent with recent studies on the implications of Covid-19 on food security and livelihoods in Nigeria [19], Kenya and Uganda [20], and in Ethiopia’s capital city of Addis Ababa [21]. Drawing on evidence from Nigeria, many households with low-income status worried about being food insecure as a result of COVID-19 policies such as lockdowns instituted by the government [19]. Accordingly, we support this idea; one of the critical themes that emerged in our study was that participants expressed a great grief of worry about having no or little access to income and other resources following the imposition of the COVID-19 preventive measures. Our finding agrees with a recent study conducted in Malawi which revealed that almost 87.78% of the population were afraid and worried of going hungry as a result of COVID-19 outbreak while expressing no concern about catching the virus [22]. Our study shows that residents who are engaged in informal and unstable sources of income—such as street vendors, sex workers and minibus drivers—were badly hit by these measures to earn reasonable amount of income. As a result, they struggle to provide adequate food for their families during the pandemic period. Most of the participants were unable to work and do other business activities as usual for their daily livelihoods due to the restrictive COVID-19 policy measures such as banning close contact, closing bars and nightclub sand marketplaces, shutting down schools, and reducing the number of passengers riding in minibuses. These policy measures provided a hostile environment for socio-economic activities by disrupting normal business activities, thereby increasing the vulnerability of the residents to food insecurity. Our findings are also consistent with a previous study about the geographies of food access which revealed that informal workers in urban areas of Blantyre were more vulnerable to food insecurity as a result of their poor or low socio-economic status [8]. Some teachers from private schools reported to have lost their jobs or were not being paid during the school closure, and this had serious implications on their daily livelihoods. Thus, sex workers, vendors, and minibus drivers faced similar fates, as their income levels dwindled due to disruption of the business activities. Given that the urban life depends on income, lack of financial and other resources has resulted in severe complications for the informal workers to afford buying adequate nutritious food for health as well as deteriorating their livelihoods.

We also agree with a study conducted in Ethiopia on COVID-19 and food security as it revealed that closure of marketplaces, income loss as well as an increase in food prices during the pandemic period brought negative consequences on the general livelihoods of people in the country [21]. Furthermore, findings of this study show people developed coping strategies to accommodate food consumption and access challenges amidst the COVID-19 pandemic. Participants have altered the normal eating sequences, especially those who used to eat three times a day to either two or once per day. The coping strategies also include consumption of less nutritious food. These findings concur with a recent study conducted in Kenya and Uganda on the impact of COVID-19 on food security, which revealed that households adjusted their dietary pattern by consuming less diversified food, reducing the usual amount of the food consumed, and skipping meals [20].

While the coping strategies contain the challenges of food consumption and access, they have devastating impacts on people's nutritional health. Skipping meals, eating less than the normal size, and consuming less nutritious food can result in malnutrition, which increases risk for illness. Thus, advancement in such coping strategies puts the lives of some Blantyre residents, especially the poor, at substantial risk of suffering from deficiency diseases, stunted growth, and increased deaths. Our claim accordingly aligns with the study on the “Early Food Insecurity Impacts of COVID 19” researched in Vermont, United States [23] despite the differences in the geographical context between these two countries. The study projected increased healthy related diseases if the Vermont population continues consuming poor dietary food as coping strategies [23]. Likewise, the COVID-19 outbreak exacerbated food insecurities among the urban dwellers in Blantyre city.

## Conclusion

Our study shows that the COVID-19 policy measures, put in place to avert the spread of the virus, have ironically brought devastating implications on the already vulnerable people for them to access basic needs such as adequate food and other means of livelihoods in selected urban areas of Blantyre in Malawi. Some of these policy measures include closure of schools, early shutdown, and closures of nightclubs/bars as well as reducing the capacity of passengers boarding minibuses or taxis. Using our understanding of ecological theory, the implementation of these COVID-19 policy measures by the authorities has heavily affected the socio-economic activities of some Blantyre residents and their livelihoods in general. Our study reveals lowering in the income levels of the already vulnerable residents whose means of livelihoods are unstable or unreliable. The vulnerable residents as identified in the study included sex workers, minibus drivers, private school teachers, and street vendors. Closure of schools deprived the private school teachers from receiving salaries while some even lost their jobs. These challenges paralyzed Blantyre residents from securing adequate and nutritional food for their families, rendering them food insecure. When respondents were asked if they had access to adequate food, most of them overwhelmingly expressed worry as well as an inability to access or buy enough food to feed their families due to having little or no money. This has forced participants to eat less nutritious food as well as skip meals thereby risking their health, safety, and well-being. Despite Malawi’s historical record of being vulnerable to food insecurity, we validate our understanding that COVID-19 policy measures have further aggravated food insecurities among the urban poor. We therefore suggest that the Malawi government should continue and expand its provisional cash transfer program by targeting the most vulnerable population in urban areas so as to alleviate the risk of food insecurity while containing further spread of the virus. There is also a need for the government to engage all stakeholders and work collaboratively with people at local levels in making and adjusting far-reaching policymaking decisions.

## Data Availability

Considering the nature of the study as it contains some sensitive information from vulnerable groups such as sex workers, therefore, the raw data will not be publicly shared following the guidelines for ethics. However, for scholars who want to validate the study, contact the corresponding author, and follow necessary procedure for data request.

## References

[1] Food Security and COVID-19 [Internet]. World Bank. [cited 2021 Nov 14]. Available from: https://www.worldbank.org/en/topic/agriculture/brief/food-security-and-covid-19.

[2] Schroeder K, Smaldone A (2015). Food Insecurity: A Concept Analysis: Food Insecurity. Nurs Forum.

[3] COVID-19 will double number of people facing food crises unless swift action is taken | World Food Programme. [cited 14 Nov 2021]. Available from: https://www.wfp.org/news/covid-19-will-double-number-people-facing-food-crises-unless-swift-action-taken.

[4] Coronavirus may push 150 million people into extreme poverty: World Bank. Reuters. 2020 [cited 14 Nov 2021]; Available from: https://www.reuters.com/article/us-imf-worldbank-poverty-idUSKBN26S2RV.

[5] World Bank Confirms Economic Downturn in Sub-Saharan Africa, Outlines Key Polices Needed for Recovery. World Bank. [cited 14 Nov 2021]. Available from: https://www.worldbank.org/en/news/press-release/2020/10/08/world-bank-confirms-economic-downturn-in-sub-saharan-africa-outlines-key-polices-needed-for-recovery.

[6] Overview. World Bank. [cited 10 Oct 2021]. Available from: https://www.worldbank.org/en/country/malawi/overview.

[7] Overview. World Bank. [cited 14 Nov 2021]. Available from: https://www.worldbank.org/en/country/malawi/overview.

[8] Riley L (2020). Malawian urbanism and urban poverty: geographies of food access in Blantyre. J Urban: Int Res Placemaking and Urban Sustainability.

[9] Boyce, C. and Neale, P. Conducting In-Depth Interview: A Guide for Designing and Conducting In-Depth Interviews for Evaluation Input.2006 Pathfinder International Tool Series, Monitoring and Evaluation-2. http://www.pathfind.org/site/DocServer/m_e_tool_series_indepth_interviews.pdf?docID=6301.

[10] Reporter N. Sex workers, minibus drivers protest Covid-19 restrictions | NewsDay. [cited 10 Oct 2021]. Available from: https://www.newsday.mw/index.php/2021/01/22/sex-workers-minibus-drivers-protest-covid-19-restrictions/.

[11] Chilanga E, Collin-Vézina D, MacIntosh H, Mitchell C, Cherney K (2020). Prevalence and determinants of malaria infection among children of local farmers in Central Malawi. Malar J.

[12] Nagawa M, Kirabira P, Atuhairwe C, Mugisha Taremwa I. Socio-Ecological Model factors influencing Fruit and Vegetable consumption among adolescents in Nakawa division, Kampala Capital City Authority, Uganda. Prev Med Commun Health [Internet]. 2018 [cited 10 Oct 2021];1(3). Available from: https://www.oatext.com/socio-ecological-model-factors-influencing-fruit-and-vegetable-consumption-among-adolescents-in-nakawa-division-kampala-capital-city-authority-uganda.php.

[13] Alemu F, Kumie A, Medhin G, Gebre T, Godfrey P. A socio-ecological analysis of barriers to the adoption, sustainablity and consistent use of sanitation facilities in rural Ethiopia. BMC Public Health. 2017;17:706. Available from: https://www.ncbi.nlm.nih.gov/pmc/articles/PMC5598066/. Cited 10 Oct 2021. 10.1186/s12889-017-4717-6 PMC5598066 28903743

[14] Informal Sector. WageIndicator subsite collection. [cited 14 Nov 2021]. Available from: https://mywage.org/malawi/labour-law/domestic-work/informal-sector.

[15] Kirchherr J, Charles K. Enhancing the sample diversity of snowball samples: Recommendations from a research project on anti-dam movements in Southeast Asia. PLoS One. 2018 [cited 10 Oct 2021];13(8):e0201710. Available from: https://www.ncbi.nlm.nih.gov/pmc/articles/PMC6104950/. 10.1371/journal.pone.0201710 PMC6104950 30133457

[16] Palinkas LA, Horwitz SM, Green CA, Wisdom JP, Duan N, Hoagwood K (2015). Purposeful sampling for qualitative data collection and analysis in mixed method implementation research. Adm Policy Ment Health.

[17] Coates J, Swindale A, Bilinsky P. Household Food Insecurity Access Scale (HFIAS) for Measurement of Food Access: Indicator Guide. Am Psychol Assoc. 2007;3:576842013–001. 10.1037/e576842013-001.

[18] Li Y, Liu G, Egolet RO, Yang R, Huang Y, Zheng Z. Knowledge, Attitudes, and Practices Related to COVID-19 Among Malawi Adults: A Community-Based Survey. Int J Environ Res Public Health. 2021;18(8):4090. Available from: https://www.ncbi.nlm.nih.gov/pmc/articles/PMC8069913/. cited 14 Nov 2021. 10.3390/ijerph18084090 PMC8069913 33924451

[19] Balana BB, Oyeyemi MA, Ogunniyi AI, Fasoranti A, Edeh H, Aiki J, et al. The effects of COVID-19 policies on livelihoods and food security of smallholder farm households in Nigeria: Descriptive results from a phone survey. 0 ed. International Food Policy Research Institute. Washington. 2020 [cited 14 Nov 2021]. Available from: https://ebrary.ifpri.org/digital/collection/p15738coll2/id/134179.

[20] Kansiime MK, Tambo JA, Mugambi I, Bundi M, Kara A, Owuor C. COVID-19 implications on household income and food security in Kenya and Uganda: Findings from a rapid assessment. World Dev. 2021;137:105199. Available from: https://www.ncbi.nlm.nih.gov/pmc/articles/PMC7500897/. cited 14 Nov 2021. 10.1016/j.worlddev.2020.105199 PMC7500897 32982018

[21] Hirvonen K, de Brauw A, Abate GT. Food Consumption and Food Security during the COVID‐19 Pandemic in Addis Ababa. Am J Agric Econ. 2021;ajae.12206. 10.1111/ajae.12206. 10.1111/ajae.12206 PMC8013419 33821007

[22] CovidFears. prezi.com. [cited 10 Oct 2021]. Available from: https://prezi.com/i/ffmbdzpiuou0/covidfears/.

[23] Niles MT, Bertmann F, Belarmino EH, Wentworth T, Biehl E, Neff R. The Early Food Insecurity Impacts of COVID-19. Nutrients. 2020;12(7):2096. Available from: https://www.ncbi.nlm.nih.gov/pmc/articles/PMC7400862/. 10.3390/nu12072096 PMC7400862 32679788

